# Serum apelin levels and cardiovascular diseases

**DOI:** 10.14744/nci.2021.33427

**Published:** 2022-05-11

**Authors:** Lutfu Askin, Husna Sengul Askin, Okan Tanrıverdi, Ali Gokhan Ozyildiz, Hakan Duman

**Affiliations:** 1 Department of Cardiology, Adiyaman Training and Research Hospital, Adiyaman, Turkiye; 2 Department of Infectious disease, Adiyaman Training and Research Hospital, Adiyaman, Turkiye; 3 Department of Cardiology, Recep Tayyip Erdogan University Faculty of Medicine, Rize, Turkiye

**Keywords:** Apelin, cardiomyocytes, inotropy, myocardial performance

## Abstract

Apelin is a G protein-linked receptor endogenous ligand, synthesized as a 77-amino acid pre-propeptide. Increased expression of apelin is present in many cardiovascular (CV) tissues, including cardiomyocytes. It is a peripheral vasodilator and one of the most potent stimulants of ventricular contraction. Apelin may be a valuable therapeutic for both blood pressure regulation and myocardial performance. More information is needed for the CV pathophysiology of apelin. We will discuss the importance of apelin level in CV diseases in this review.

## Highlight key points

- Apelin decreased in patients with the severe LV dysfunction, and low apelin levels predict the survival of STEMI patients.
- Apelin has a positive effect on the CV system through a series of reactions, such as vasodilation, contraction, and degradation of inflammation.
- Apelin level is independently associated with LVH in essential hypertensive patients.
- Apelin may be a novel biomarker for determining the severity and development of coronary atherosclerosis.
- Apelin is a promising therapeutic target for ischemic heart disease.

Apelin is originated from white adipose tissue and binds to G protein as a ligand [1, 2]. Apelin appears to be a promising adipocytokine for cardiovascular diseases (CVDs). Since there is no review in the literature about this subject, we aim to focus on the prognostic significance of apelin levels in CVD.

## THE ROLE OF SERUM APELIN LEVELS IN ST ELEVATION MYOCARDIAL INFARCTION

Apelin is a potent inotrope besides vasodilation [3]. Low apelin level is one of the causes of heart failure (HF); in a study with rats, it has been shown to protect myocardial damage caused by isoproterenol [4–6].

Apelin is a promising therapeutic target for ischemic heart disease. Liu et al. [7] reported that serum apelin level might be used as an essential predictor of post-PCI prognosis in ST-elevation myocardial infarction (STEMI). Apelin is a component of CV homeostasis [8–10]. In two studies conducted in Poland, plasma apelin levels were lower in STEMI patients and it was insufficient to predict adverse events [11, 12].

Among STEMI, those with low apelin levels have poor clinical profiles. The MACE incidence is also higher in these patients [13]. Apelin decreased in patients with the severe left ventricular (LV) dysfunction, and low apelin levels predict the survival of STEMI patients [4, 14]. The relationship of apelin and morbidity proves that it can be an independent predictor of prognosis [7].

Apelin causes vasodilatation and plays a role in inflammation [14]. Studies showed that apelin prevented the formation of the aortic aneurysm by its direct anti-inflammatory effect [15]. Besides, apelin limits oxidative stress, the main component of myocardial reperfusion injury. Apelin prevents reperfusion damage by reducing reactive oxygen species (ROS) and affecting superoxide dismutase enzyme [16–18]. Furthermore, apelin is an inotropic agent [19].

## THE RELATIONSHIP BETWEEN APELIN AND CONTRAST-INDUCED NEPHROPATHY IN PATIENTS WITH CONGESTIVE HF

**Figure 1:**
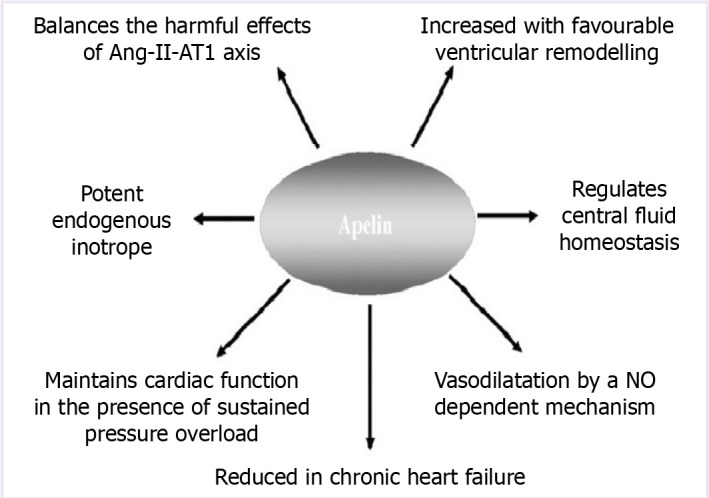
Positive effects of apelin in heart failure.

The effect of the contrast agent to cause nephropathy is through ROS and vasoconstriction [20]. Apelin, a recently discovered cytokine, has several effects on the CV system. In addition to antioxidant and anti-inflammatory activity, apelin plays a role in homeostatic balance by its vasodilator and inotropic effects (Fig. 1) [21–23]. Apelin causes vasodilation by releasing endothelium-derived nitric oxide (NO) [24].

Apelin causes potent heart contraction [25]. In studies with rats, cardiac contraction has been impaired after a decrease in apelin level [26]. Besides, apelin causes ventricular remodeling heart tissue, and its level is increased in HF. Apelin regulates fluid homeostasis by affecting the hypothalamus and pituitary [21]. Short peptide of apelin regulates the CV system, and longer sequence peptides of apelin regulate the immune system [27].

In recently published experiments, apelin has been shown to reduce cytochrome C release and caspase-3 activation in neuron cultures as well as ROS and mitochondria depolarization. Apelin-13 acts as an endogenous cell-protective cytokine capable of inhibiting excitotoxic death and apoptosis in neurons [28]. In studies of atherosclerosis and diabetes models, apelin increases NO bioavailability by reducing reactive ROS production [29].

## APELIN LEVELS IN CORONARY ARTERY DISEASE (CAD)

Increased expression of apelin is present in cardiomyocytes, CV tissues, vascular smooth muscle, and endothelial cells. Apelin has a positive effect on the CV system through a series of reactions, such as vasodilation, contraction, and degradation of inflammation. The anti-atherogenic property of apelin in humans is still unproven [30].

Some researchers suggested that apelin reduction is associated with coronary atherosclerosis. Apelin may be a novel biomarker for determining the severity and development of coronary atherosclerosis, but extensive studies should support this result [26].

## ARTERIOVENOUS FISTULA TYPE, NYHA CLASS, AND APELIN LEVELS IN HEMODIALYSIS (HD) PATIENTS

Arteriovenous fistula affects both systolic and diastole in the heart. Fistula may cause HF by increased cardiac output. Apelin was significantly higher in NYHA Class I–II patients than NYHA Class III–IV. The relationship between apelin and LV end-diastolic diameter in hemodialysis patients supports this finding [31].

Apelin is released from the endothelium in the CV system [32]. Malyszko et al. [31] showed that von Willebrand factor is associated with apelin. After myocardial damage, the release of apelin increases from endothelial cells [33]. Małyszko et al. [34] reported that apelin affects cardiac functions in HD patients. As a result, apelin decreases in dialysis patients with CAD, and its level is associated with cardiac functions. Apelin is involved in the pathogenesis of CAD in chronic renal failure (CRF). Due to the inotropic properties of apelin, it may be useful clinically in uremic cardiomyopathy.

Low apelin levels have not yet been disclosed in patients with CRF. Catabolism of cellular proapelin and/or excessive apelin release may cause decreased apelin levels. Hemodynamic factors such as hypervolemia or hyperkinetic circulation may contribute to these findings [27].

## APELIN IN HYPERTROPHIC CARDIOMYOPATHY (HCM)

HCM characterized by sarcomeric protein mutation causes sudden death in young. Hypertrophy and fibrosis are the two causes of all adverse events in HCM [35, 36]. Plasma apelin levels correlate with the degree of late gadolinium increase (LGE) in HCM patients. The effect of lower apelin level on fibrosis has not been elucidated yet in HCM. Immunocytochemistry analysis has shown that apelin is exposed in cardiomyocytes, vascular, and endocardial endothelium [37]. The atrium and epicardial adipose tissues are the main resource of apelin. Furthermore, only apelin-13 was shown as the main isoform in human in spectral analysis [38].

Cardiac, renal, and pulmonary artery fibrosis are associated with decreased apelin expression. Cardiac fibrosis often accompanies HF. Apelin rises in the early phase of HF, but progressively reduces during the disease period. In HCM, reduced apelin levels were detected in LGE-positive patients due to atrial fibrosis [39].

## SERUM APELIN AND BICUSPID AORTIC VALVE (BAV)

BAV is one of the common congenital diseases. While it has a benign course in childhood, valve dysfunction may develop in advanced ages. BAV cause not only the aortic valve involvement but may also cause enlargement of thoracic aorta [40, 41].

While BAV is frequently accompanied by valvular diseases, concurrent aortic pathologies such as aneurysm and dissection may be present in some patients. The mechanisms that cause these different manifestations are not clear. Unidentified interactions at the molecular level may be responsible for these differences.

After intravenous administration of apelin-13 to rats, decrease in blood pressures (BP) was observed [42]. Tatemoto et al. [24] showed that NO mediated the vasoactive effects of apelin. Endothelial NO synthase release disruption is the cause of aortic dilatation [43]. Low serum apelin level was observed in the BAV regardless of the aortic diameter. Apelin level reduction may cause aneurysm formation in BAV [43].

## THE RELATIONSHIP OF SERUM APELIN LEVELS WITH LV HYPERTROPHY (LVH) IN HYPERTENSION

Increased cardiomyocyte size and fibrosis in the extracellular matrix are pathological changes in LVH [44]. Ye et al. [45] reported that serum apelin level is independently associated with LVH in essential hypertensive patients. Furthermore, apelin may be the therapeutic target to improve blood pressure regulation and cardiac function.

## APELIN AND CALCIFIC AORTIC STENOSIS

Aortic valve stenosis (AS) is one of the most common heart diseases, with an annual mortality rate of approximately 14%. Associated structural diseases complicate the treatment of AS. Different factors are related to the onset and progression of AS [46].

Duman et al. [47] showed decreased apelin levels and increased hs-CRP concentrations in patients with severe calcific AS. Their findings may help clarify the pathophysiological role of apelin in CVD.

## Conclusion

Recent studies emphasize that apelin is a potent vasodilator and an inotropic agent. Apelin reduces myocardial damage caused by myocardial infarction. Due to its inotropic properties, it may be a new treatment option for patients with HF. Little evidence exists that apelin is an independent predictor for CVD. Extensive studies should support the data to accept that apelin determinates the severity and development of coronary atherosclerosis.
